# Study on the Deterioration of Concrete under Dry–Wet Cycle and Sulfate Attack

**DOI:** 10.3390/ma13184095

**Published:** 2020-09-15

**Authors:** Fang Liu, Tonghuan Zhang, Tao Luo, Mengzhen Zhou, Kunkun Zhang, Weiwei Ma

**Affiliations:** Shaanxi Key Laboratory of Safety and Durability of Concrete Structures, Xijing University, Xi’an 710123, China; liufang_winter@163.com (F.L.); 525748333dian@sina.cn (T.Z.); mengzhen865580934@126.com (M.Z.); 13892267707@163.com (K.Z.); 18829298831@163.com (W.M.)

**Keywords:** concrete, dry–wet cycle, sulfate attack, compressive strength, pore structure

## Abstract

In order to study the deterioration and mechanism of dry–wet cycles and sulfate attack on the performance of concrete in seaside and saline areas, the deterioration of compressive strength of concrete with different water cement ratios under different erosion environments (sodium sulfate soaking at room temperature and coupling of dry–wet cycling and sodium sulfate) was studied here. At the same time, ICT (industrial computed tomography) and NMR (nuclear magnetic resonance) techniques were used to analyze the internal pore structure of concrete under different erosion environments. The results show that the compressive strength under different erosion environments increases first and then decreases, and the dry–wet cycle accelerates the sulfate erosion. With the increase of dry and wet cycles, larger pores are filled with erosion products and developed into small pores in the early stage of erosion; in the later stage of erosion, the proportion of larger pores increases, and cracks occur inside the sample. In the process of sulfate soaking and erosion, the smaller pores in the concrete account for the majority. As the sulfate erosion continues, the *T*_2_ spectrum distribution curve gradually moves right, and the signal intensity of the larger pores increases.

## 1. Introduction

Concrete has become the most widely used engineering material due to its advantages of fast setting and hardening, low price, and good durability [1,2,3,4,5]. However, during the service of the concrete structure, the adverse effects of its own material defects and the complexity of the environment on the structure will result in concrete failure before the service life [6].

There are high concentrations of sulfate in the soil and groundwater in coastal areas, saline soil areas, acid rain coverage areas [7]. Concrete structures in areas with water level fluctuation, wave splashing, and tidal range are exposed to long-term sulfate erosion with alternating wet and dry conditions [8]. Two impregnants, pure-silane and water-based silane, were taken as treatment and applied on concrete samples for protecting from harmful saline environments by Al-Kheetan et al. [9].

Sulfate corrosion is one of the main factors which affect the durability of concrete and the coupling between dry–wet cycles, and sulfate shows strong harm and complex mechanisms in corrosion of concrete. Therefore, it is of great significance to carry out research on the deterioration of concrete under the coupling of sulfate erosion and the dry–wet cycle. Many researchers have made a lot of achievements in the study of sulfate-eroded concrete [10,11,12,13,14]. But due to the differences in the simulated experimental environment [15,16,17,18], the selected test method [19,20,21], and the concrete mix ratio used in the test [22,23], etc., up to now, there is no unified test system.

The appearance of concrete deterioration is the reduction of macro mechanical properties, while the reason for deterioration is the change of internal structure. At present, with regard to the mechanism research of sulfate eroding concrete, micro-analysis mainly for the composition and microstructure of erosion products is by technical means such as SEM [24,25,26], XRD [27,28], etc. However, there are few studies on the evolution of internal meso-pore characteristics. Since the pore structure is one of the main factors affecting the strength of concrete, it is very important to study the changes of the pores in the concrete during the erosion process.

In this paper, the deterioration of concrete with different mix ratios is considered under two erosion environments (sodium sulfate soaking at room temperature, and coupling of dry–wet cycling and sodium sulfate). The macroscopic performance of sulfate-eroded concrete is characterized by the compressive strength; at the same time, the evolution of the internal pore structure of concrete after sulfate-eroded is analyzed by ICT and NMR techniques.

## 2. Materials and Methods

### 2.1. Materials

Ordinary Portland 42.5R cement was used in this experiment. Its chemical composition and main physical and mechanical properties are shown in Table 1 and Table 2, respectively.

Natural river sand with fineness module of 2.8 was used as fine aggregate. Natural crushed stones with continuous grading between 5 and 20 mm and bulk density of 1720 kg/m^3^ were used as coarse aggregates. The fly ash was produced by Weihe Power Plant in Shaanxi, China. Its chemical composition and physical properties are shown in Table 3 and Table 4, respectively.

The polycarboxylate superplasticizer was produced by Shandong Yousuo Chemical Technology Co., Ltd. The technical parameters are shown in Table 5. The water used for mixing concrete was ordinary tap water in Xi’an, China. The sodium sulfate used in the test was produced by Yatai United Chemical Co., Ltd., Wuxi, China, with the molecular weight of 142.04.

### 2.2. The Mix Ratio of Concrete

Water to cement (W/C) ratios of 0.50 and 0.45 were adopted in this test, as shown in Table 6.

Two water to cement ratios, two erosion environments, and four different erosion ages were considered. Three samples were prepared for compressive test under each condition. Forty-eight samples were prepared in total.

After pouring, the concrete specimens stood for 24 h and were then demolded. All specimens were put into a standard curing box for 28 days, with the temperature of 20 ± 2 °C and a relative humidity of 95%.

### 2.3. Experimental Design

#### 2.3.1. The Erosion Environment

The cured samples were divided into two groups. One group was immersed in Na_2_SO_4_ solution at room temperature (Group J), and the other group was under the coupling environment of dry–wet circulation and Na_2_SO_4_ solution erosion (Group D). The Na_2_SO_4_ solution in both groups had a mass fraction of 5%. During the erosion process, the solution was replaced every 30 days to prevent the concentration change of Na_2_SO_4_.

**Figure 1:**
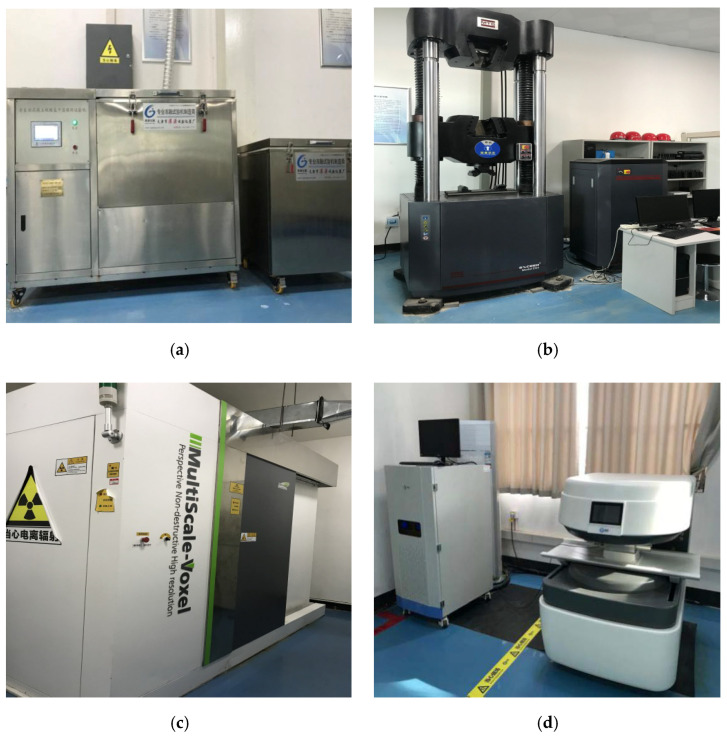
Test instruments and equipment: (**a**) The fully automatic machine for dry–wet circulation of sulfate; (**b**) MTS universal testing machine; (**c**) ICT; (**d**) The MacroMR 12-150H-I instrument.

The treatment of Group D samples was carried out by a fully automatic machine for dry–wet circulation of sulfate, as shown in Figure 1a. According to GB/T 50082-2009, the treatment process is as follows:

(1) Immerse the cured sample in sodium sulfate solution for 15 h at a temperature of 25 °C.
(2) Discharge the solution immediately after soaking and then air dry for 1 h.
(3) Dry the sample at a temperature of 80 °C for 6 h.
(4) After that, the samples were cooled by air for 1.5 h, and finally cooled for 0.5 h, with the cooling temperature of 25 °C.

The above steps (1)~(4) indicated a dry–wet cycle of sulfate attack (1 d) and then were repeated in sequence until the end of the test.

#### 2.3.2. Mechanical Test

For the compression test, the sample size was 100 mm × 100 mm × 100 mm. The measured values were converted according to the standard size (150 mm × 150 mm × 150 mm), and the size conversion factor is 0.95. The universal testing machine of MTS, with the maximum test force of 2000 kN, was used here as shown in Figure 1b. The reference test standard is GB/T 50081-2002.

The relative value of concrete compressive strength (*K_c_*) is defined in this paper:

(1) $$K_{c}=\frac{f_{cc}{}^{n}}{f_{cc}{}^{0}},$$

where *f_cc_^n^* is the compressive strength of concrete after sulfate attack (MPa); *f_cc_*^0^ is the compressive strength of concrete without sulfate attack (MPa).

#### 2.3.3. Industrial CT test

In this paper, industrial CT (MS-Voxel450, Tianjin, China, as shown in Figure 1c) was used to explore the internal structure of concrete, which mainly consists of X-ray source, X-ray imaging detector, precision sample stage, image acquisition system, image analysis and processing software system, 3D image reconstruction and processing system, etc. Avizo software was used to obtain information such as the pore size distribution and pore number of the sample.

In order to investigate the changes of the internal pore characteristics of concrete after dry–wet cycles and sulfate attack, ICT and post-processing software (Avizo) were adopted in this paper to obtain the distribution of pores in the concrete. By comparison of the pore structure changes in the process of erosion, the effect of dry–wet cycles and sulfate attack on the pore evolution of concrete was analyzed.

#### 2.3.4. Nuclear Magnetic Resonance Test

The NMR test is mainly to detect the hydrogen nuclei in concrete, and gives the *T*_2_ spectrum distribution curve, which can reflect the internal pore distribution of concrete.

In this paper, the NMR testing instrument (MacroMR 12-150H-I, Suzhou, China) was used, as shown in Figure 1d. The magnetic field strength of the permanent magnet is 0.3 T, the pulse frequency range is 1~30 MHz, and the magnet temperature is 32 °C.

## 3. Test Results and Analysis of Compressive Strength

**Figure 2:**
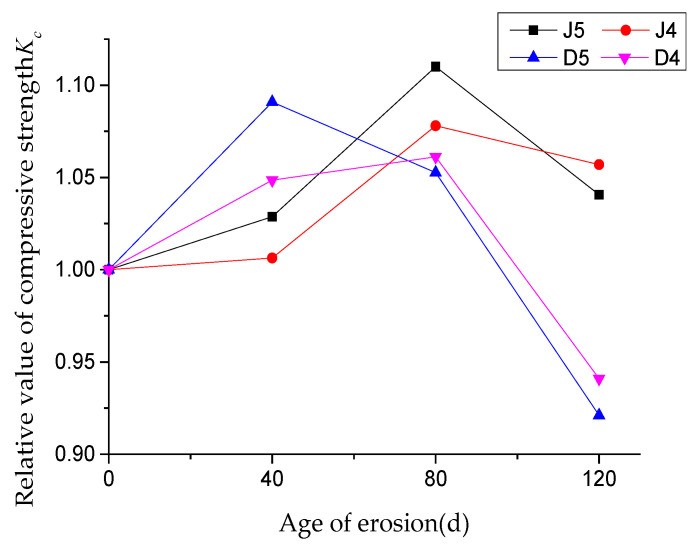
The relationship between the relative compressive strength and erosion age.

The test results of compressive strength of concrete with different water–cement ratios under different erosion periods are shown in Table 7, and the relationship between the relative compressive strength Kc and the age of erosion is shown in Figure 2. J5 and J4 respectively represent concrete samples soaked in Na_2_SO_4_ solution at room temperature with a water–cement ratio of 0.5 and 0.45, while D5 and D4 represent concrete samples under coupling of dry–wet circulation and Na_2_SO_4_ solution erosion with water–cement ratio of 0.5 and 0.45, respectively. For each erosion age, the compressive strength was the average value of 3 samples.

It can be seen from Table 7 and Figure 2 that the compressive strength shows a trend of increasing first and then decreasing. The compressive strength of group J reaches the peak when immersed in Na_2_SO_4_ solution for 80 days, and the growth rate of group J5 is faster than that of group J4. The compressive strength of groups J5 and J4 at the peak increases to 111.0% and 107.8% of that of the uneroded concrete, respectively. Group D5 reaches the peak value after 40 dry–wet cycles of sulfate attack, while group D4 reaches the peak strength after 80 dry–wet cycles of sulfate attack. Compared with the uneroded samples, the peak compressive strength of D5 and D4 groups increases by 9.1% and 6.1%, respectively.

As the age of erosion continues to increase, the compressive strength of concrete decreases at different rates. Compared with the peak compressive strength, after 120 days of erosion, the J5 and J4 groups respectively decrease by 6.3% and 2.0%, while the D5 and D4 groups decrease by 15.6% and 11.3%, respectively. It indicates that the effect of dry and wet circulation on the compressive strength of concrete is greater, and concrete with a larger water–cement ratio is more sensitive to sulfate attack.

According to the curves of relative compressive strength of concrete in Figure 2, it can be seen that with the increase of erosion days, concrete with different water–cement ratios shows a similar trend, which is mainly divided into two stages. In the first stage, the increasing of compressive strength at different rates, may be due to the Na_2_SO_4_ solution penetrating into the concrete and forming corrosion products by the chemical reaction, which can fill the internal pores of concrete, and thus improve compressive strength. In the second stage, the compressive strength of concrete begins to decline, which may be caused by the continuous generation of corrosion products (ettringite and gypsum) squeezing the pore wall, accelerating the formation and development of cracks in concrete, and then resulting in the decreasing of compressive strength. Sulfate erosion products (ettringite and gypsum) are expansionary, with the increase of erosion age, erosion products increase, gradually fill and squeeze the pores inside the concrete, and finally may produce cracking. This caused the effect of ettringite and gypsum was at its maximum after 80 days of erosion.

By comparing the results of J5 and D5, it can be seen that the compressive strength of J5 rises and falls at a relatively slow rate, while in group D5, the compressive strength decreases obviously after a short period of strength increase. It shows that dry–wet cycles make damages in concrete accumulate repeatedly and accelerate the deterioration of concrete.

## 4. Analysis on Pore Development in Concrete

### 4.1. ICT Results of Concrete under the Action of Dry–Wet Cycles and Sulfate Attack

#### 4.1.1. Pore Characteristics of Concrete

**Figure 3:**
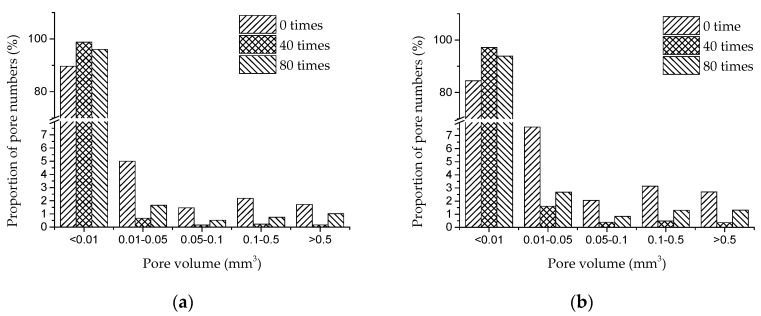
Proportion of pore numbers in concrete under dry and wet cycles of sulfate attack: (**a**) D5; (**b**) D4.

**Figure 4:**
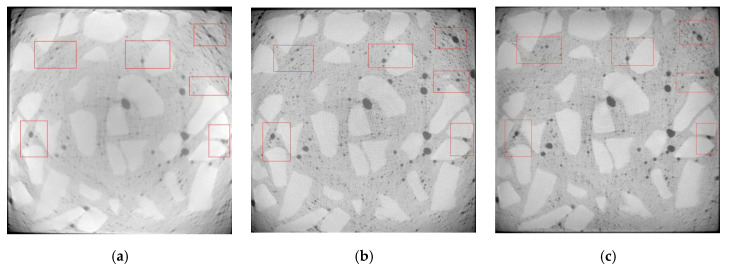
CT images of D5 under different dry and wet cycles of sulfate attack: (**a**) 0 dry–wet cycle; (**b**) 40 dry–wet cycles; (**c**) 80 dry–wet cycles.

**Figure 5:**
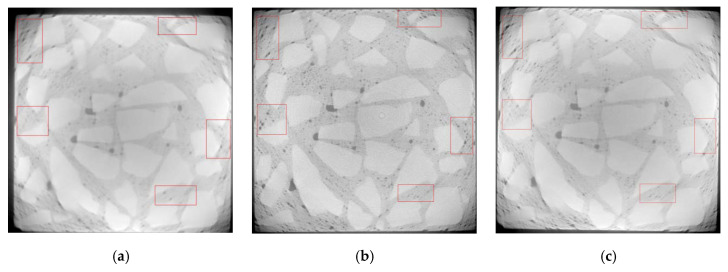
CT images of D4 under different dry and wet cycles of sulfate attack: (**a**) 0 dry–wet cycle; (**b**) 40 dry–wet cycles; (**c**) 80 dry–wet cycles.

Referring to [29], pores in concrete are divided into five ranges according to the pore volume. Figure 3 shows changes in the proportion of pore numbers in concrete under dry and wet cycles of sulfate attack. Figure 4 and Figure 5 respectively show the evolution of pores in D5 and D4 under the action of dry–wet cycles and sulfate attack.

It can be seen from Figure 3 that the pores in concrete are unevenly distributed, and the number proportion of pores in the range of 0~0.05 mm^3^ is significantly more than that of larger pores.

As shown in Figure 3, with the increase of dry–wet cycles, the number proportion of pores in the range of 0~0.01 mm^3^ increases first and then decreases, while the proportion of pores larger than 0.01 mm^3^ shows a trend of decreasing first and then increasing.

It is known that the sulfate solution permeates into the concrete through the pores on the surface and forms insoluble substances by the chemical reaction with hydration products [30]. With the larger surface area, the SO_4_^2−^ in larger pores may react more fully with hydration products, and more erosion products will be generated in larger pores, which may promote larger pores evolving into small ones. At the same time, the continuous hydration reaction may also generate new smaller pores inside. Therefore, in the early stage of sulfate attack (0 to 40 dry–wet cycles), the proportion of pores in the range of 0~0.01 mm^3^ increases, while the proportion of pores larger than 0.01 mm^3^ is significantly reduced. Generally, compressive strength of concrete is improved in this period.

As sulfate attack continued, some pores in concrete are filled with erosion products. Hydration products are continuously consumed, which results in the decreasing of adhesion between aggregates. On the other hand, erosion products continuously generated have dilatability. The expansion force and crystallization pressure may induce cracking in concrete, and cracks will further expand and develop with the erosion of sulfate. Therefore, in the later stage of sulfate attack (40 dry–wet cycles to 80 dry–wet cycles), the proportion of pores in the range of 0 to 0.01 mm^3^ decreases, and the proportion of pores larger than 0.01 mm^3^ increases. Concrete with larger water–cement ratio (D5) shows decreasing of compressive strength in this period.

#### 4.1.2. Analysis of Crack Growth in Concrete

The selection of CT images starts from the top of concrete. The surface and inner layers of D5 are located at about 5 and 25 mm, respectively, while the surface and inner layers of D4 are located at about 8 and 30 mm, respectively.

**Figure 6:**
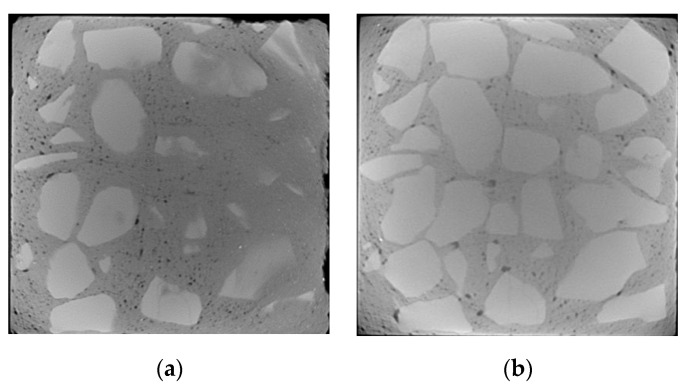
CT images of D5 before sulfate attack (no cracks): (**a**) surface layer; (**b**) inner layer.

**Figure 7:**
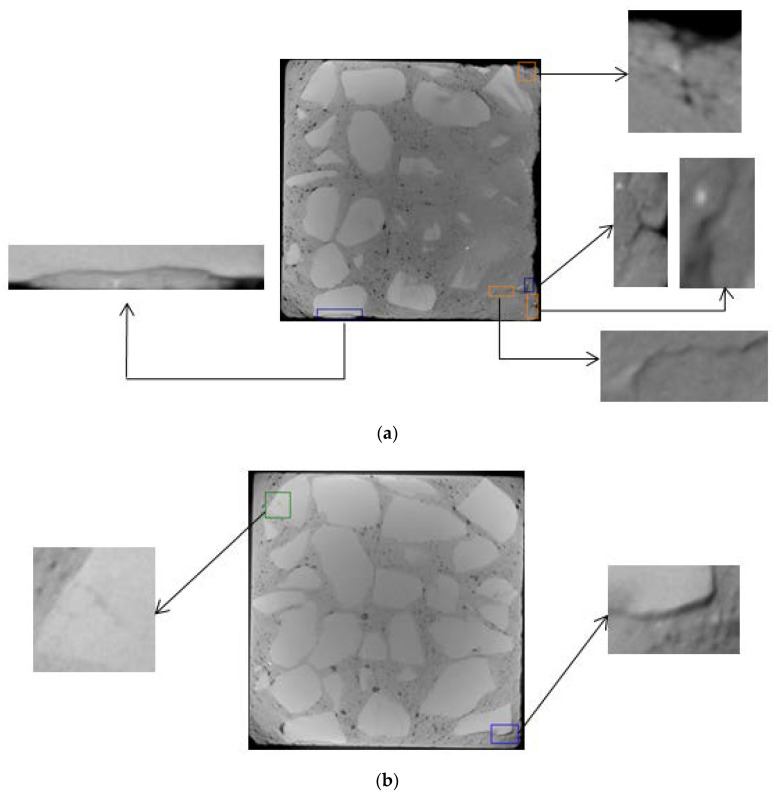
Crack propagation images of D5 under 80 dry–wet cycles of sulfate attack: (**a**) surface layer; (**b**) inner layer.

**Figure 8:**
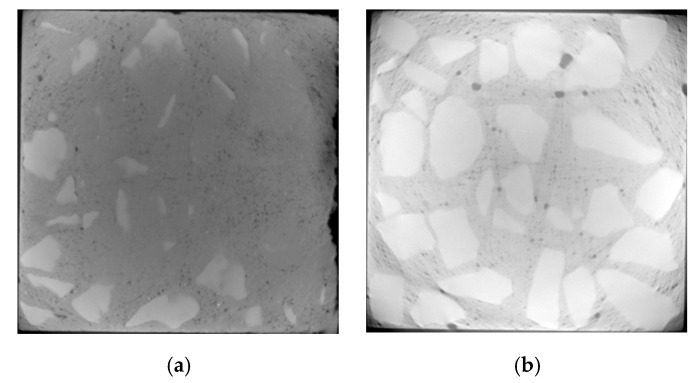
CT images of D4 before sulfate attack (no cracks): (**a**) surface layer; (**b**) inner layer.

**Figure 9:**
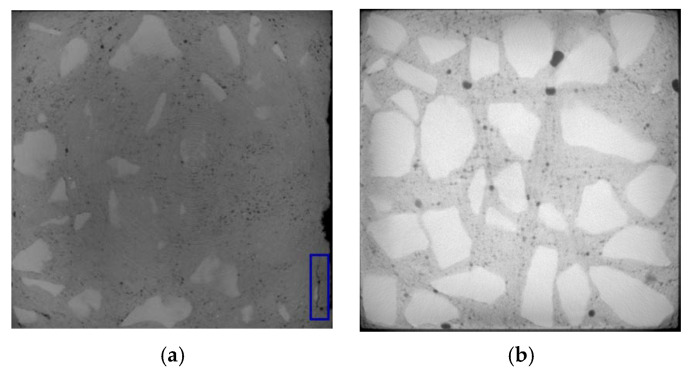
Crack propagation images of D4 under 80 dry–wet cycles of sulfate attack: (**a**) surface layer; (**b**) inner layer.

Under the action of dry and wet cycles, sulfate reacts physically and chemically with the internal components of concrete. The generated erosion products cause cracking on the surface and interior of the concrete. There are three main types of cracks: mortar cracks, aggregate cracks, and aggregate–mortar interface cracks, which are indicated by yellow, green, and blue in Figure 6, Figure 7, Figure 8 and Figure 9, respectively.

From Figure 6, Figure 7, Figure 8 and Figure 9, it can be seen that after 80 dry–wet cycles of sulfate attack, several cracks with different sizes appear on the surface and inner layers of D5, as shown in Figure 7; an aggregate–mortar interface crack appears at the corner of D4, as shown in Figure 9a.

From the comparison between Figure 6 and Figure 7, it can be seen that there are more cracks on the surface layer of concrete, mainly distributed on the edge of concrete. From the surface layer to inner layer, the number of cracks gradually decreases, mainly composed of the aggregate crack and aggregate–mortar interface crack. With the erosion of sulfates, cracks in concrete continue to occur and develop, causing damage to the concrete structure, and then the compressive strength of D5 decreases. From the comparison between Figure 7 and Figure 9, it is found that cracks in D4 are less than that in D5 after 80 dry and wet cycles of sulfate attack, and cracks in D4 are only on the surface layer, which indicate that concrete with lower water–cement ratio has better resistance to sulfate corrosion. The lower the water–cement ratio, the denser the interior and the fewer pores of concrete, thus the sulfates have less erosion of concrete through the pores.

### 4.2. NMR Test Results of Concrete Soaked in Na_2_SO_4_ Solution at Room Temperature

#### 4.2.1. T_2_ Spectrum Distribution of Pore Fluid

**Figure 10:**
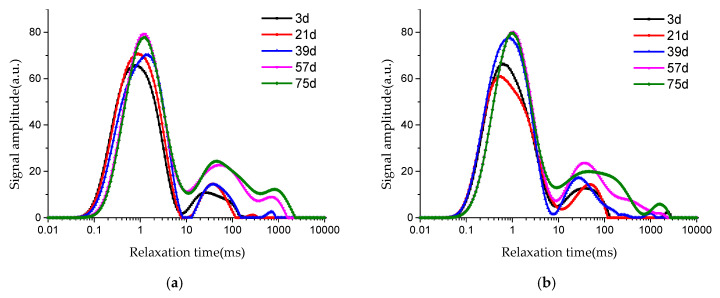
*T*_2_ spectrum distribution of concrete soaked in Na_2_SO_4_ solution: (**a**) J5; (**b**) J4.

Figure 10 shows the *T*_2_ spectrum distribution curve of pore fluid in concrete under soaking and erosion of Na_2_SO_4_ solution. The relaxation time on the horizontal axis reflects the radius of pore fluid. The larger relaxation time corresponds to the larger pore fluid radius.

From the shape of *T*_2_ spectrum distribution curves in Figure 10, it can be seen that there is a main peak and 1~3 sub-peaks. The main peak is much higher than the sub-peak, which indicates that the proportion of smaller pore fluid in concrete is much more than that of larger pore fluid. With the increase of soaking time in Na_2_SO_4_ solution, *T*_2_ spectrum distribution curves of J5 and J4 move to the right, that is, moving in the direction of the larger pore size, and the signal intensity of larger pores generally shows an increasing trend, which reveals the corrosion of sodium sulfate gradually penetrating into the interior of concrete.

When concrete is soaked in Na_2_SO_4_ solution for 57 days, the third peak of *T*_2_ spectrum curves of J4 has no significant change, while the third peak of J5 changes obviously, as shown in Figure 10. Based on this, it can be speculated that concrete with lower water–cement ratio has better resistance to sulfate corrosion, in which the proportion of larger pores is less.

#### 4.2.2. *T*_2_ Spectrum Area of Pore Fluid

**Figure 11:**
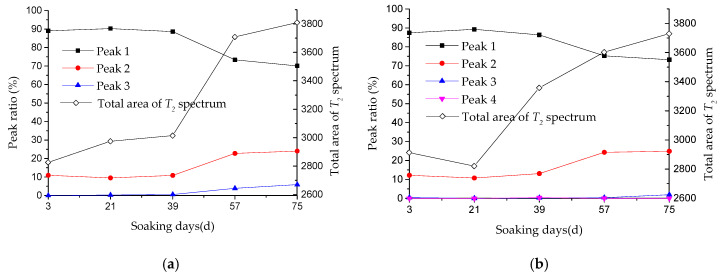
*T*_2_ spectrum area of concrete under sodium sulfate erosion: (**a**) J5; (**b**) J4.

The area enclosed by the *T*_2_ spectrum distribution curve reflects the hydrogen content inside the sample, that is, the pore fluid content of concrete. Table 8 and Figure 11 give *T*_2_ spectrum area and peak proportion of pore fluid in concrete under sodium sulfate immersion and erosion.

As shown in Table 8 and Figure 11, with the increase of soaking time in Na_2_SO_4_ solution, the total area of *T*_2_ spectrum shows an increasing trend in general, which reflects the increasing of pore volume in concrete to some extent.

When concrete is immersed in Na_2_SO_4_ solution for 21 days, the total area of *T*_2_ spectrum for J4 decreases. The reason for this may be that SO_4_^2−^ in sodium sulfate solution reacts with hydration products in concrete and then insoluble substances are generated, which fill larger pores, causing peak 3 in *T*_2_ spectrum to disappear. During the immersion in sodium sulfate solution (3 to 57 d), proportions of peak 3 and 4 (larger pores) for J4 are very small.

From Table 8, it can be seen that peak 1 accounts for more than 70% of the total area, and the area proportion of peak 2 is between 9% and 25%. The proportion of peak 3 and 4 is in the range of 0~6%, which indicates that the pore distribution in concrete is mainly composed of small pores (peak 1).

As shown in Figure 11, we can see that during the period of sulfate attack from 3 to 39 d, the area proportion of peak 1 and peak 2 remains basically stable. At the same time, compared with test results in Table 7, it can be found that the compressive strength of Group J at 0 and 40 d erosion age also has no obvious change.

During the period of sulfate attack from 39 to 57 d, the area proportion of peak 1 and peak 2 changes greatly. Compared with the value of 39 d, the area proportion of peak 1 and peak 2 for the J5 group respectively increases by −17.2%, 110.0%, while for the J4 group it increases by −12.7% and 85.9%, respectively. It may be due to the erosion products filling small pores and then reducing small pore volume. The pore walls are subject to expansion, causing the small pores to evolve into larger ones and then increasing the larger pore volume significantly. It can be found that the pore structure of concrete with larger water cement ratio has more significant changes.

As sulfate attack continues, the proportion of peak 3 increases gradually, which respectively reaches 3.87% and 5.87% at 57 and 75 d for the J5 group, and reaches 1.92% at 75 d for the J4 group. In the early erosion, the proportion of peak 3 is relatively small, which indicates the change of pore structure is dominated by the development of smaller pores (peak 1 and peak 2).

## 5. Conclusions

The objective of this research is to study the deterioration of dry–wet cycles and sulfate attack on the performance of concrete, the deterioration of compressive strength of concrete with different water cement ratios under different erosion environments (sodium sulfate soaking at room temperature and coupling of dry–wet cycling and sodium sulfate) was studied. At the same time, ICT (industrial computed tomography) and NMR (nuclear magnetic resonance) techniques were used to analyze the internal pore structure of concrete under different erosion environments. Some main conclusions can be drawn as following:

(1) Under the two erosion environments of group D and group J, the compressive strength of concrete shows the similar trend of first increasing and then decreasing, and the dry–wet cycles accelerate sulfate corrosion.
(2) According to ICT results, the number proportion of pores within the range of 0~0.01 mm^3^ in group D accounts for the most, which increases first and then decreases with the increase of dry–wet cycles. In the early erosion, larger pores in concrete are filled with erosion products and develop into small pores. In the later stage of erosion, the proportion of small pores decreases, while the proportion of larger pores increases, and cracks appear inside concrete.
(3) After 80 dry–wet cycles, obvious cracks appear on the surface and inner layers of D5 with larger water cement ratio. Cracks on the surface layer of concrete, mainly distributed on the edge of concrete. From the surface layer to inner layer, the number of cracks gradually decreases.
(4) Under soaking and erosion of Na_2_SO_4_ solution, the *T*_2_ spectrum distribution of pore fluid for group J is dominated by peak 1, and the main peak is much higher than the sub-peak, which indicates that the proportion of smaller pore fluid in concrete is in the majority. With the increase of soaking time in Na_2_SO_4_ solution, the *T*_2_ spectrum distribution curve gradually moves toward the larger pore size, and the signal intensity of larger pores generally shows an increasing trend.

## Figures and Tables

**Table 1:** Chemical composition of ordinary Portland cement (%).

SiO_2_	Fe_2_O_3_	Al_2_O_3_	CaO	MgO	SO_3_	Alkali	Loss on Ignition
22.81	3.36	5.62	61.43	1.35	2.17	0.54	2.60

**Table 2:** Physical and mechanical properties of ordinary Portland cement.

Density (g/cm^3^)	Specific Surface Area (m^2^/kg)	Fineness (%)	Setting Time (min)		Compressive Strength (MPa)		Flexural Strength (MPa)	
			Initial Setting	Final Setting	3 Days	28 Days	3 Days	28 Days
3.10	360	3.8	125	180	24.0	50.5	4.7	8.3

**Table 3:** Chemical composition of fly ash (%).

SiO_2_	Al_2_O_3_	Fe_2_O_3_	CaO	MgO	Na_2_O, K_2_O
49.02	31.56	6.97	4.88	0.83	1.78

**Table 4:** Physical properties of fly ash (%).

Fineness (45μm)	Water Requirement Ratio	Loss on Ignition	Water Content	SO_3_
18	94	3.65	0.3	1.2

**Table 5:** Technical parameters of superplasticizer.

Appearance	Hydroxyl	PH	Moisture	Solubility
Light yellow to white flake	22~27	5.0~7.0	≤0.5	Soluble in water and various organic substances

**Table 6:** Mixing ratio of concrete samples.

W/C	W/CM	Cement (kg/m^3^)	Fly ash (kg/m^3^)	Sand (kg/m^3^)	Coarse Aggregate (kg/m^3^)	Water (kg/m^3^)	Superplasticizer (kg/m^3^)
0.50	0.402	330	80	806	1026	165	7.6
0.45	0.375	365	75	776	1031	165	8.1

**Table 7:** Compressive strength of concrete under different erosion ages.

Label	0d (MPa)	40d (MPa)	80d (MPa)	120d (MPa)
J5	41.8 ± 1.1	43.0 ± 1.3	46.4 ± 3.6	43.5 ± 1.8
J4	47.4 ± 0.8	47.7 ± 0.4	51.1 ± 1.4	50.1 ± 3.0
D5	41.8 ± 1.1	45.6 ± 1.9	44.0 ± 4.8	38.5 ± 1.2
D4	47.4 ± 0.8	49.7 ± 1.2	50.3 ± 3.2	44.6 ± 1.9

**Table 8:** *T*_2_ spectrum area and peak proportion of pore fluid.

Label	Soak Days (d)	Total Area of *T*_2_ Spectrum	The Proportion of Peaks (%)			
			Peak 1	Peak 2	Peak 3	Peak 4
J5	3	2826.39	89.09	10.91	\	\
	21	2974.90	90.27	9.50	0.24	\
	39	3014.17	88.64	10.85	0.52	\
	57	3708.11	73.36	22.78	3.87	\
	75	3809.68	70.09	24.04	5.87	\
J4	3	2913.03	87.45	12.16	0.38	\
	21	2820.78	89.25	10.75	\	\
	39	3357.62	86.29	13.08	0.26	0.38
	57	3602.63	75.31	24.31	0.35	0.03
	75	3729.39	73.21	24.87	1.92	\
